# Epidemiological Trend of Sepsis in Patients with Hospital Admissions Related to Hepatitis C in Spain (2000–2015): A Nationwide Study

**DOI:** 10.3390/jcm9061607

**Published:** 2020-05-26

**Authors:** Alejandro Alvaro-Meca, Irene Maté-Cano, Pablo Ryan, Verónica Briz, Salvador Resino

**Affiliations:** 1 Department of Preventive Medicine & Public Health, Rey Juan Carlos University, 28933 Alcorcón, Madrid, Spain; Alejandro.alvaro@urjc.es; 2 Laboratory of Reference and Research in Viral Hepatitis, National Centre for Microbiology, Institute of Health Carlos III, 282220 Majadahonda, Madrid, Spain; irmate@hotmail.com; 3 Primary Care Center “Ensanche de Vallecas”, SERMAS, 28051 Madrid, Spain; 4 Servicio de Medicina Interna, Hospital Universitario Infanta Leonor, 28031 Madrid, Spain; pabloryan@gmail.com; 5 Instituto de Investigación Sanitaria Gregorio Marañón (IiSGM), 28009 Madrid, Spain

**Keywords:** hepatitis C, sepsis, hospital admission, mortality, epidemiology, hospital resources

## Abstract

Background: Hepatitis C virus (HCV) infection predisposes patients to other infectious diseases, such as sepsis. We aimed to analyze epidemiological trends of sepsis-related admissions, deaths, and costs in hospital admissions with chronic hepatitis C who had a hospital admission in Spain. Methods: We performed a retrospective study of all hospitalizations involving chronic hepatitis C in the Spanish Minimum Basic Data Set (MBDS) between 2000 and 2015. This period was divided into four calendar periods (2000–2004, 2005–2007, 2008–2011, and 2012–2015). Results: We selected 868,523 hospital admissions of patients with chronic hepatitis C over 16 years in the Spanish MBDS. Among them, we found 70,976 (8.17%) hospital admissions of patients who developed sepsis, of which 13,915 (19.61%) died during admission. We found an upward trend, from 2000–2003 to 2012–2015, in the rate of sepsis-related admission (from 6.18% to 10.64%; *p* < 0.001), the risk of sepsis-related admission (from 1.31 to 1.55; *p* < 0.001), and the sepsis-related cost per hospital admission (from 7198€ to above 9497€; *p* < 0.001). However, we found a downward trend during the same study period in the sepsis case-fatality rate (from 21.99% to 18.16%; *p* < 0.001), the risk of sepsis-related death (from 0.81 to 0.56; *p* < 0.001), and the length of hospital stay (LOHS) (from 16.9 to 13.9; *p* < 0.001). Moreover, the rate of bacterial Gram-positive and candidiasis infections decreased, while Gram-negative microorganisms increased from 2000–2003 to 2012–2015. Conclusions: Sepsis, in chronic hepatitis C patients admitted to the hospital, has increased the period 2000–2015 and has been an increasing burden for the Spanish public health system. However, there has also been a significant reduction in lethality and LOHS during the study period. In addition, the most prevalent specific microorganisms have also changed in this period.

## 1. Introduction

The World Health Organization (WHO) has estimated that there are around 71 million individuals with chronic hepatitis C worldwide, many of whom are unaware of their hepatitis C virus (HCV) infection [1]. Spain had one of the highest HCV infection prevalences in Europe in 2013 [2], with values around 1.5% for HCV antibodies and 1.2% for plasma HCV-RNA. However, this situation has changed after the appearance of the direct-acting antivirals (DAAs) with a prevalence of antibodies against HCV of 0.8% and active HCV infection of 0.22% [3].

Chronic hepatitis C causes persistent liver inflammation, leading to the development of cirrhosis in approximately 10–20% of patients after 20–30 years of infection. Cirrhotic patients have an annual risk of hepatocellular carcinoma (HCC) around 1–5% and a 3–6% annual risk of hepatic decompensation. After an episode of decompensation, the risk of death in the following year is between 15% and 20% [4]. Moreover, chronic hepatitis C causes a substantial economic cost on the National Health Services in Europe, mainly due to the management of patients with advanced liver disease stages [5,6,7,8].

HCV-infected patients are also predisposed to other infectious diseases [9,10,11], possibly because it shares transmission routes with these pathogens or due to the dysregulation of the immune system that appears during chronic hepatitis C, particularly in the advanced stage of cirrhosis [12,13]. Alvaro-Meca et al. (2016) found that HCV-infected patients with decompensated cirrhosis have a higher incidence of sepsis than patients with compensated cirrhosis admitted to the intensive care unit (ICU) [14].

**Figure 1:**
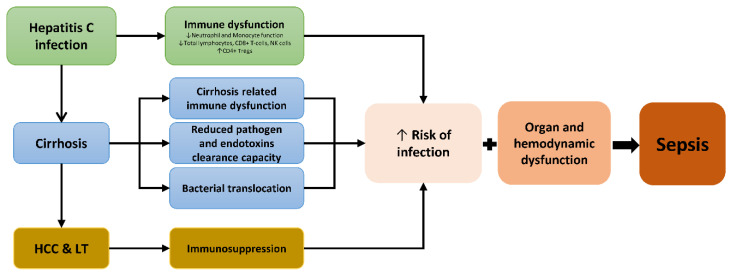
Summary of the relationship among hepatitis C infection, cirrhosis, immune system, bacterial infection, and sepsis. Abbreviations: HCC, hepatocellular carcinoma; LT, liver transplantation; NK, natural killer.

HCV causes immune dysfunction [15], which could increase the vulnerability to bacterial infections regardless of cirrhosis, in particular, sepsis and lower respiratory tract, reproductive, and urinary tract infections [16] (Figure 1). Cirrhosis, per se, increases the risk of infection by causing neutrophil and macrophage dysfunction, an increase of proinflammatory cytokines, bacterial translocation, and a decreased clearance of endotoxins [12,13,17] (Figure 1). This pathophysiological process can trigger cirrhosis-associated immune dysfunction (CAID) in decompensated cirrhosis, accompanied by bacterial infections and sepsis. [12,13] (Figure 1).

HCV treatment has changed substantially in the last years [18]. Until 2012, pegilated (peg)-interferon(IFN)/ribavirin (RBV) was the only treatment option for chronic HCV infection in Spain, with a very low rate of sustained virological response SVR (40–55%) [19]. Then, in 2011, the first generation of DAAs appeared (boceprevir, telaprevir, and simeprevir), which were used with peg-IFN/RBV, reaching response rates between 65–85% [19]. Finally, in 2014, the second generation of DAAs (such as sofosbuvir, ombitasvir, daclatasvir, ledipasvir, and paritaprevir among others) became available in combination with peg-IFN-α/RBV, reaching response rates between 80–95%, and in IFN-free therapies, with SVR rates >95% [19]. Furthermore, all-oral DAAs treatments are safer and shorter in duration than previous IFN-based therapies.

Sepsis is described as a life-threatening dysfunction of the organs caused by a dysregulated response to infection by the host [20]. Sepsis is the primary cause of death from infection, especially if not recognized and treated promptly, affecting millions of people worldwide. [21,22,23]. The rise in the incidence of sepsis has promoted global efforts to improve its diagnosis and management, which has reduced intra-hospital deaths from sepsis worldwide [24]. Thus, the case-fatality rate (CFR) has decreased in most developed countries [25,26,27], although the CFR of sepsis remains higher than other relevant pathologies such as cancer and Acquired Immune Deficiency Syndrome (AIDS) [28].

The incidence of sepsis fluctuates across the world, and in most developed countries, sepsis has progressively increased during the last decades [23]. Sepsis is also a substantial economic burden worldwide because patients with sepsis require a high expenditure on hospital resources, and the costs of sepsis are quite high [26,29].

### Objective

Both chronic hepatitis C infection and sepsis are major health problems worldwide. Since hepatitis C infection increases the risk of bacterial infections and sepsis, improvements in HCV treatment might have an impact on sepsis. In this study, we aimed to analyze the epidemiological trend of sepsis-related admissions, deaths, and costs in hospital admission with chronic hepatitis C who had a hospital admission during the 2000–2015 period in Spain.

## 2. Methods

### 2.1. Study Design

We carried out a nationwide population-based retrospective study of all hospitalizations involving chronic hepatitis C in the Spanish Minimum Basic Data Set (MBDS) between 1 January 2000 and 31 December 2015.

### 2.2. Data Source

Data were obtained from the MBDS of the Ministry of Health, Consumption, and Social Welfare (MHCSW). The MBDS is a database that contains epidemiological and clinical data recorded at the time of hospital discharge: identification number (encrypted), dates of birth, sex, hospital admissions and discharge, up to 14 diagnosis codes according to the International Classification of Diseases, 9th ed., Clinical Modification (ICD-9-CM), and outcome at discharge. All these data are anonymized, making it impossible to verify if a patient was admitted more than once during the time considered. However, it is possible to identify various admissions of the same patient in the same hospital. The Spanish MHCSW sets strict standards for the maintenance of the MBDS and performs periodic checks. The MBDS covers around 92% of hospital discharges registered in Spanish hospitals, 84% in public hospitals and 16% in private hospitals [30]. The National Health System provides free medical care to 99.5% of the Spanish population.

### 2.3. Ethics Statement

The Spanish MBDS is regulated by the Law of Spain, which requires personal data related to the health of patients to Spanish hospitals. This transferred database was anonymized, so it was not necessary to obtain informed consent from patients. The MHCSW revised our protocol of investigation and confirmed that our study fulfilled all ethical considerations according to Spanish legislation. Our study was approved by the Research Ethics Committee (Comité de Ética de la Investigación y de Bienestar Animal) of the Instituto de Salud Carlos III (Madrid, Spain) (Ref.: P36_2019-v2).

### 2.4. ICD-9-CM Codes and Patients

The ICD-9-CM codes used in this study are shown in Supplemental Table S1. The included hospital admissions were coded in the MBDS with a diagnosis of chronic hepatitis C (ICD-9-CM: 070.44, 070.51, 070.54, 070.70, 070.71, or V02.62). Hospital admissions with hepatitis B were excluded. Then, we selected those patients that had sepsis (presence of bacterial or fungal infections and organ dysfunction). Namely, we selected all hospitalizations with ICD-9-CM codes for bacterial and fungal infections (ICD-9-CM codes used by Angus et al. [21]; see Supplementary Table S1) and a diagnosis of acute organ dysfunction (ICD-9-CM codes used by Angus et al. [21], Dombrovskiy et al. [31], and Shen et al. [32]; see Supplementary Table S1). 

We also explored specific microorganisms in the hospital admissions with a sepsis diagnosis (see Supplementary Table S1), among which are: (i) Gram-positive (+): staphylococci (coagulase-negative staphylococci and *Staphylococcus aureus*), streptococci, enterococci; (ii) Gram-negative (−): *Escherichia coli*, *Pseudomonas*, *Klebsiella*, *Haemophilus influenzae*, *Serratia*; and (iii) Fungal infection: candidiasis, aspergillosis, and zygomycosis. We only showed microorganisms with a prevalence higher than 1%.

### 2.5. Main Study Variables

The primary outcome variables were: (1) sepsis-related admission: hospital admission with sepsis; (2) sepsis-related death: in-hospital-death among hospital admission with sepsis; (3) length of hospital stay (LOHS): number of days that patients spend in hospital; and (4) sepsis-related costs: cost related to hospital admissions. We also analyzed the presence of codes for specific microorganisms related to sepsis (see Supplementary Table (ST) S1).

The main study factor was the time stratified into four calendar periods: (a) from 2000 to 2003 (2000–2003); (b) from 2004 to 2007 (2004–2007); (c) from 2008 to 2011 (2008–2011); and (d) from 2012 to 2015 (2012–2015).

### 2.6. Statistical Analyses

The rate of sepsis-related admissions was calculated as the number of hospital admissions with sepsis and chronic hepatitis C divided by all hospital admissions coded in the MBDS with a diagnosis of chronic hepatitis C. In the same way, CFR was estimated as the proportion of hospitalized chronic HCV-infected patients with sepsis that died. The prevalence and CFR of specific microorganisms was calculated as the ratio between the number of times a particular microorganism was found and the number of hospital admissions or those patients with hospital admission who died. All rates were expressed in percentages.

The LOHS was calculated as the difference between the date of discharge or death and the date of hospital admission. Hospital discharge on the same day was considered to be a one day stay. Sepsis-related costs were calculated using diagnosis-related groups (DRG) data extracted from the MBDS [30], and adjusting by the inflation increment of that same period in Spain.

Categorical data and proportions were analyzed using the chi-squared test or Fisher’s exact test, as required. Continuous variables were analyzed by the Kruskal–Wallis test. The temporal trend was evaluated using the Extended Mantel Haenszel Chi-Square for linear trend for categorical variables and Mann–Kendall Trend Test for continuous variables in Y values.

We also calculated the odds ratio (OR) of sepsis-related admission and sepsis-related death, according to the calendar period, by using logistic regression models adjusted by the main clinical and epidemiological covariates. The risk of sepsis was adjusted by gender, age, urgent admission, surgical condition, Charlson index, and liver disease severity. The risk of sepsis-related death was adjusted by gender, age, urgent admission, surgical condition, Charlson index, length of stay, liver disease severity, number of acute organ dysfunction, organism-specific sepsis, and site of infection.

Statistical analysis was performed using the R statistical package version 3.1.1 (GNU General Public License) [33]. All tests were two-tailed with *p*-values < 0.05 considered significant.

## 3. Results

### 3.1. Study Population

**Figure 2:**
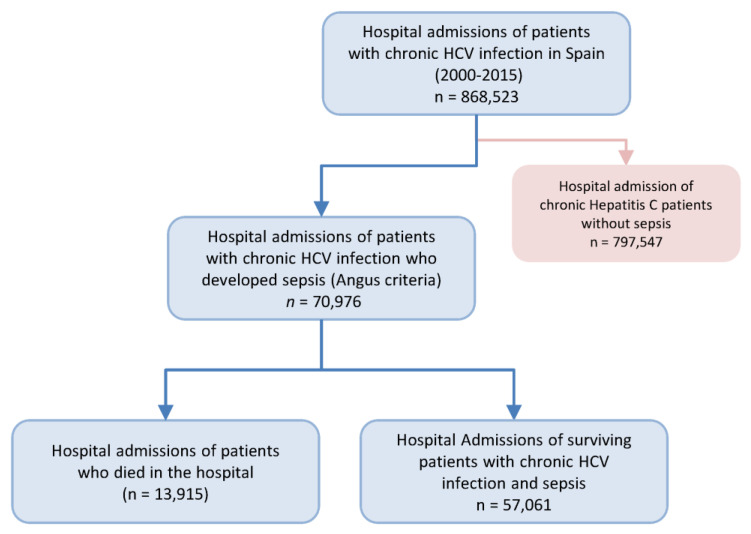
Flow chart of the selection of hospital admissions, who were included in this study, with chronic hepatitis C and sepsis in Spain (1997 to 2014).

Overall, 868,523 hospital admissions of patients with chronic HCV infection over 16 years were selected in the Spanish MBDS during the study period (2000–2015). Among them, we found 70,976 (8.17%) hospital admissions of patients who developed sepsis, of which 13,915 (19.61%) died in the hospital (Figure 2).

Table 1 shows the characteristics of hospital admissions of patients with hepatitis C and sepsis. Overall, most were men (>60%) with a median age of around 60 years, and drugs were the most frequent substance of abuse. The vast majority had an urgent admission (>90%) and a Charlson co-morbidity index higher than 4. End-stage liver disease was the most frequent liver-related clinical event. As for the characteristics related to sepsis, we found that more than half of hospital admissions only had one acute organ dysfunction, being kidney and lung the most frequent. The respiratory system was the most frequent site of infection.

### 3.2. The Trend of Sepsis-Related Admission and Sepsis-Related Death

**Figure 3:**
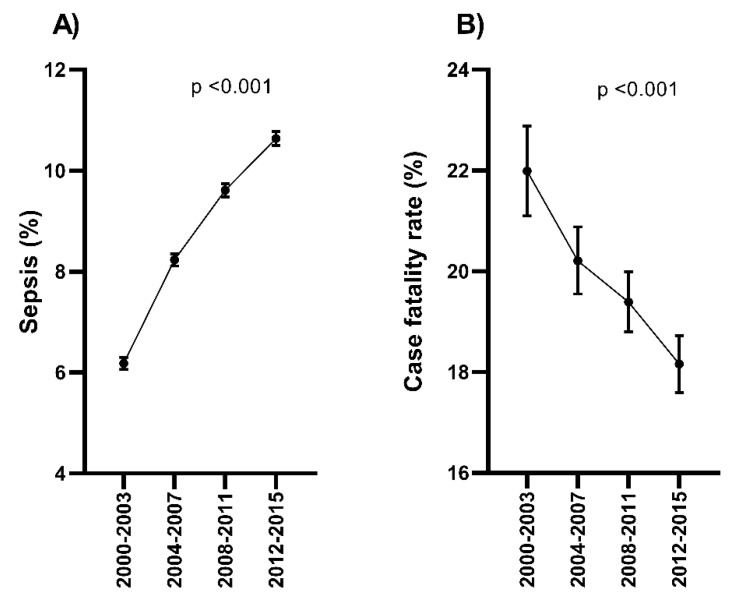
Temporal trend of the sepsis rate (regarding all hospital admissions with a diagnosis of chronic HCV infection, %) and the sepsis-related death (regarding chronic HCV-infected patients with (**A**) hospital admission and sepsis, (**B**) CFR, %) in Spain (2000–2015). Statistic: Values were expressed as percentages. The Extended Mantel Haenszel Chi-Square was used to calculate the linear trend from 2000–2003 to 2012–2015. Abbreviations: HCV, hepatitis C virus; CFR, case-fatality rate.

We found a significant upward trend, from 2000–2003 to 2012–2015, in the rate of hospital admissions with sepsis (from 6.18% to 10.64%; *p* <0.001; Figure 3A), while the CFR of sepsis showed a significant downward trend (from 21.99% to 18.16%; *p* <0.001; Figure 3B) during the same study period (full description in Table S2).

### 3.3. Temporal Trend of the Risk of Sepsis-Related Admission and Sepsis-Related Death

**Figure 4:**
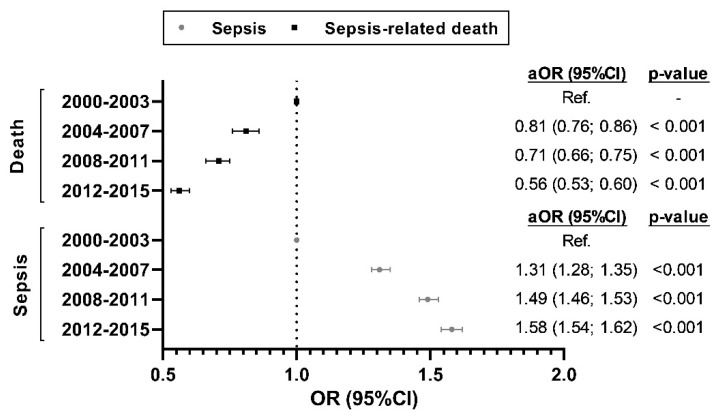
Temporal trend of the risk of sepsis (regarding all hospital admissions with a diagnosis of chronic HCV infection) and the risk of sepsis-related death (regarding chronic HCV-infected patients with hospital admission and sepsis) in Spain (2000–2015). Statistic: Values were expressed as odds ratios (OR) and 95% of confidence intervals (95%CI). *p*-values were calculated by logistic regression analysis. Abbreviations: HCV, hepatitis C virus; aOR, adjusted odds ratio; 95% CI, 95% confidence interval.

For sepsis-related admissions, the adjusted OR (aOR), using 2000–2003 as reference, had a significantly increasing trend during the whole follow-up period (from 1.31 to 1.58; *p* < 0.001). The last three calendar periods (2004–2007, 2008–2011, and 2012–2015) showed significant differences (*p* < 0.001) concerning the initial period (2000–2003) (Figure 4, full description in Table S3).

For sepsis-related death, the aOR, referring to 2000–2003, had a significant downward trend during the follow-up (from 0.81 to 0.56; *p* < 0.001), and the last three calendar periods showed significant differences (*p* < 0.001) concerning the initial period (2000–2003) (Figure 4, full description in Table S3).

### 3.4. Trends in Costs for Hospital Admission with Sepsis

**Figure 5:**
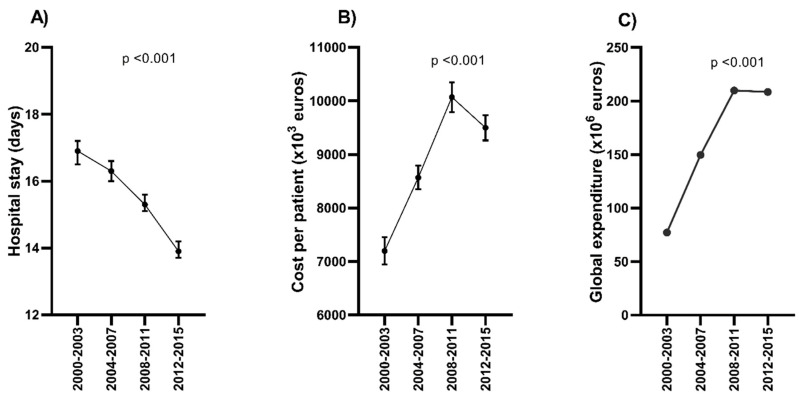
Temporal trend of (**A**) the length of hospital stay and (**B**,**C**) the cost in hospital admissions of patients chronic hepatitis C and sepsis in Spain (2000–2015). Statistic: Values expressed as mean [95% Confidence Interval (CI)]. The linear trend, from 2000–2003 to 2012–2015, was calculated by the Mann–Kendall Trend Test. Abbreviations: HCV, hepatitis C virus.

The average LOHS was 15.3 days during the whole study period. The LOHS values were lower in survivors than in non-survivors (15.3 vs. 16.1; *p* < 0.001). Furthermore, the LOHS decreased from 16.9 to 13.9 between 2000 and 2015 (*p* < 0.001), particularly after 2007 (Figure 5A, full description in Table S4).

The average hospital cost per hospital admission was 9089€ during the whole study period. Furthermore, the average hospital cost per hospital admission increased from 7198€ to above 10,000€ between 2000 and 2011 (*p* < 0.001), but then decreased to 9497€ in 2012–2015 (Figure 5B, full description in Table S4).

The average national cost for hospitalization was 645.1 M€ during the whole study period. The total expenditure increased from 77.1 M€ in 2000–2003 to over 200 M€ after 2007 (*p* < 0.001), and then it stabilized (Figure 5C, full description in Table S4).

### 3.5. Epidemiological Trends of Specific Microorganisms

Overall, the more frequent microorganisms were staphylococci among Gram (+), *Escherichia coli* among Gram (−), and Candida among fungi (see Supplementary Table S5).

**Figure 6:**
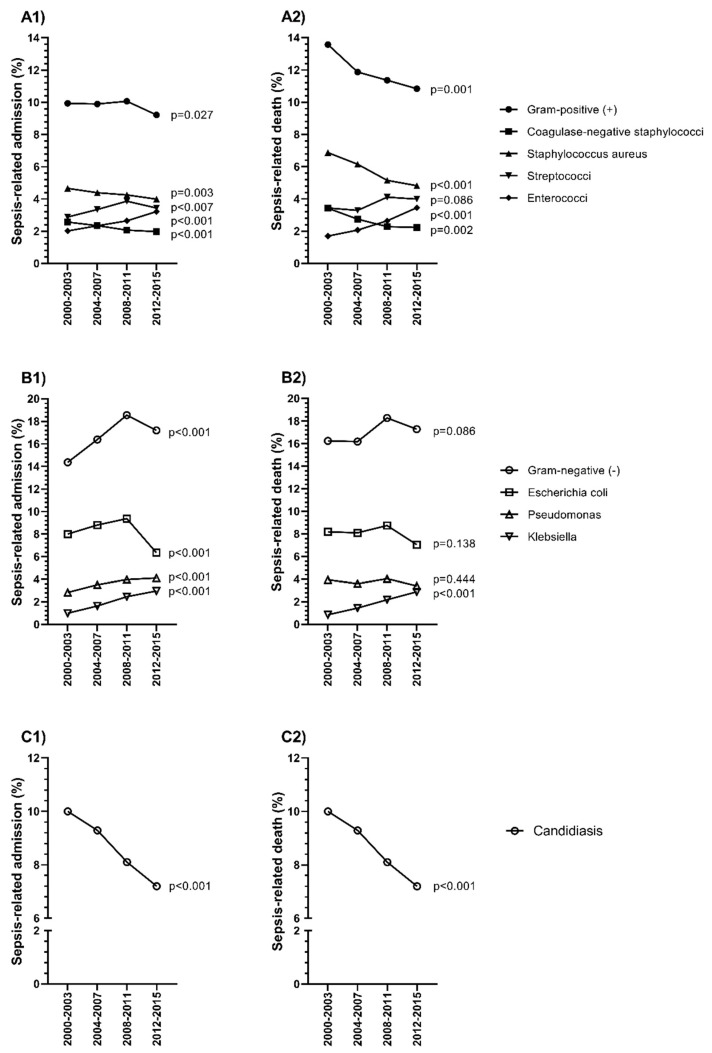
Temporal trend of microorganism specific rate (not including unknown) linked to sepsis and sepsis-related death in hospital admissions of patients with chronic hepatitis C and sepsis in Spain (2000–2015). (**A1**): Sepsis-related admission with diagnosis of Gram-positive (+); (**A2**): Sepsis-related death with diagnosis of Gram-positive (+); (**B1**) Sepsis-related admission with diagnosis of Gram-negative (−); (**B2**) Sepsis-related death with diagnosis of Gram-negative (−); (**C1**) Sepsis-related admission with diagnosis of candidiasis; (**C2**) Sepsis-related death with diagnosis of candidiasis. Statistic: Values were expressed as percentages. The Extended Mantel Haenszel Chi-Square was used to calculate the linear trend from 2000–2003 to 2012–2015. Abbreviations: HCV, hepatitis C virus.

For sepsis-related admissions, the rate of Gram positives (+) showed a slightly significant downward trend (from 9.94% to 9.22%; *p* = 0.027, Figure 6A1, full description in Table S5), while the rate of Gram-negatives (−) showed a significant upward trend (from 14.37% to 17.2%; *p* < 0.001; Figure 6B1, full description in Table S5). Within the Gram (+), we found a significant downward trend for coagulase-negative staphylococci (from 2.57% to 1.97%; *p* < 0.001) and *Staphylococcus aureus* (from 4.66% to 3.99%; *p* = 0.003). In contrast, we found a significant upward trend for Streptococci (from 2.88% to 3.44%; *p* = 0.007) and Enterococci (from 2.02% to 3.22%; *p* < 0.001). Within the Gram (−), we found a significant upward trend for *Pseudomonas* (from 2.84% to 4.11%; *p* < 0.001) and *Klebsiella* (from 1% to 2.97%; *p* < 0.001), but a significant downward trend for *Escherichia coli* (from 8% to 6.35%; *p* < 0.001) was also found. Moreover, a decrease in candidiasis was observed (from 10% to 7.2%; *p* < 0.001), (Figure 6C1, full description in Table S5).

For sepsis-related death, the rate of Gram (+) showed a significant downward trend (from 13.57% to 10.84%; *p* < 0.001; Figure 6A2, full description in Table S5), while the rate of Gram (−) was stable (from 16.24% to 17.28%; *p* = 0.086; Figure 6B2, full description in Table S5). Within the Gram (+), we found a significant downward trend for coagulase-negative staphylococci (from 3.43% to 2.23%; *p* = 0.002) and *Staphylococcus aureus* (from 6.87% to 4.82%; *p* < 0.001); while a significant upward trend was found for Enterococci (from 1.7% to 3.46%; *p* < 0.001). Within the Gram (−), a significant upward trend was only found for *Klebsiella* (from 0.85% to 2.88%; *p* < 0.001). Moreover, we found a decrease in candidiasis (from 8.31% to 5.92%; *p* < 0.001) (Figure 6C2, full description in Table S5).

## 4. Discussion

In this retrospective study of hospital admissions of patients with chronic hepatitis C and sepsis in Spain during a 16-year period, from 2000 to 2015, we found that the rate of sepsis-related admissions, risk of sepsis, and sepsis-related costs increased, while the CFR of sepsis, risk of sepsis-related death, and LOHS decreased. We also found that the rate of bacterial Gram-positive and candidiasis decreased, while Gram-negative microorganisms increased during the follow-up. To our knowledge, this is the first study that analyzes the nationwide epidemiological trend of sepsis-related admissions in the chronic hepatitis C during the 21st century (2000–2015).

In this study, we found that the rate of sepsis-related admissions and the risk of sepsis-related admissions increased during the study period. Previous studies have documented the rise in hospital admissions, both in chronic hepatitis C [5,34] and in sepsis [35,36], in the 21st century in Spain. On the one hand, the rise in hepatitis C admissions is related to the natural history of HCV infection and the extensive exposure to hepatitis C virus due to blood derivatives not tested for hepatitis C between 1970 and 1990, and the use of intravenous drug injection specially in the 1980s [37]. As these patients got older, liver disease progressed, and the burden of chronic hepatitis C disease increased [38]. Additionally, cirrhosis increases the risk of infections due to unspecific immune dysfunctions added to the increased bacterial translocation [12,13,17], and the predisposition of patients infected with HCV to develop other infectious diseases [9,10,11]. Once the infection is established, the excessive immune response plus the hemodynamic dysfunction related to cirrhosis favors the development of sepsis [16]. On the other hand, sepsis incidence and mortality have also grown worldwide in recent decades [39], mainly due to the aging of the population [36], as well as to a higher prevalence of comorbidities, a more extensive use of immunosuppressive treatments and invasive procedures, nosocomial infections related to resistant microorganisms, and a better sepsis recognition and coding [40].

In our study, we found that the CFR of sepsis and the risk of sepsis-related death decreased during the 16-year study period, which is consistent with the overall upward trend found in prior studies [36,41]. In our opinion, the decrease in CFR of sepsis is very relevant given the increase in the age, urgent admission, Charlson comorbidity index, and the number of acute organ dysfunction, all factors with a negative influence on the prognosis of patients. Furthermore, an increase in CFR has been reported in patients admitted to hospitals with chronic hepatitis C infection, regardless of the presence of sepsis [42,43,44,45,46]. Moreover, this downward trend can also be attributable to a greater awareness of the severity of sepsis and a general improvement in intensive care [24]. Additionally, coding practices could have become more inclusive due to increased awareness of sepsis during the last decade [47], which could include a higher number of less severe patients and increase the denominator for the calculation of CFR. The impact of the different antiviral treatments against HCV that have been used during the study period has to be taken into consideration (peg-IFN/ribavirin and DAAs). Although the SVR rates increased from 40% to 95% during the study period, HCV therapy has allowed reduction of the number of subjects with active HCV infection and slows down the progression of liver disease in a large percentage of the cured patients [48]. Despite these advances in HCV therapy, residual fibrosis may remain for a long time [48], and the vast majority of HCV-infected individuals remain undiagnosed and untreated, putting these patients at risk of progression to cirrhosis [49].

The LOHS is a useful measure since its reduction is a reflection of a faster recovery of patients and a reduction in hospital expenses and resources. In our study, the average LOHS was 15.4 days, which is longer than LOHS previously reported with the Spanish MBDS for general admissions of chronic HCV-infected patients (9.2 days) [5], but similar to that described for sepsis in Spain during the 21st century (15.3 days) [36]. Compared with data from other countries, there are similar differences since LOHS reported in chronic hepatitis C infected patients is around 4–7 days [46,50,51], and sepsis is between 10–15 days [21,27,52,53,54]. Therefore, patients included in the study resemble those with sepsis in the general population. Moreover, in our study, LOHS decreased during the study period, particularly after 2007. This trend is similar to that described in previous studies in the general population from Spain [36], the US [53,54,55], or Brazil [56]. In this case, in our study, the decreasing trend in LOHS is very relevant in the context of higher values of age, urgent admission, Charlson comorbidity index, and the number of acute organ dysfunction during the follow-up. As discussed above, this reduction in LOHS during the study period may be related to a general improvement in intensive care [24], more inclusive coding practices [47], and the impact of HCV treatments [19,48].

The trend of hospital costs (per patient and total) was inverse to the trend of LOHS and increased from 2000–2003 to 2008–2011, but hospital costs decreased (cost per patient) or stabilized (national expenditure) in the 2012–2015 period. Similar increases in the national expenditure of sepsis before 2012 have been found in Spain [36], the United States [53], and South Korea [57]; a comparable cost decline in the national expenditure of sepsis after 2012 was also described in Spain [36] and Brazil [56]. This decrease in hospital costs after 2011 could be due to the economic crisis [58,59,60] and the impact of other factors, such as greater adherence to treatment guidelines [25], the appearance of DAAs that increased the response rate of HCV therapy [61], and more inclusive coding practices [47].

Several studies have shown an increased risk of bacterial infection and sepsis in HCV-infected patients [14,15,62], even if they have not developed cirrhosis [10,16]. In HCV-infected patients, invasive pneumococcal disease [9] and *Staphylococcus aureus* infection [63] are among the most common bacterial infections. In cirrhotic patients, the endogen infections due to enteric Gram (−) bacteria and *Enterococcus* spp. are the leading causes of bloodstream infections. However, the improvement in patient management and the increased use of invasive procedures has increased the risk of exogenous infections, such as *Staphylococcus* spp. [64]. In sepsis, the main causative agents in the Spanish population are *Escherichia coli*, *Staphylococcus aureus*, *Enterococcus*, *Pseudomonas,* and *Klebsiella* spp. [65,66]. In our study population, the most frequent microorganisms were Gram-negative bacilli, particularly *Escherichia coli,* followed by *Staphylococci*, particularly *Staphylococcus aureus* and *Streptococci*, *Enterococci*, *Pseudomonas* sp. and *Klebsiella* sp. in lower percentages. Therefore, overall, there is not a significant difference between our findings and previously published data regarding the microbiological profile of sepsis episodes, both in the general population and in cirrhotic patients.

We also observed significant changes in sepsis-related microorganisms during the study period. On the one hand, Gram (+) showed a decrease in *Staphylococcus* and an increase in *Streptococcus* and *Enterococcus* during the study period. On the other hand, Gram (−) showed a decrease in *Escherichia coli* and an increase in *Pseudomonas* and *Klebsiella*, particularly in sepsis-related admissions. In this regard, an overgrowth of potentially pathogenic bacteria in the gut has been reported in cirrhotic patients, predisposing to the development of infections in these patients [67]. However, the abundance of *Enterobacteriaceae*, *Enterococcus,* and *Staphylococcus* in the gut microbiota in HCV-infected patients with cirrhosis decreases after HCV clearance with antiviral therapy [68], which could change the risk of infection.

Moreover, we found a high rate of candidiasis during the whole study period. Mucosal candidiasis is a limited tissue invasion, but once the microorganism penetrates the mucosal/skin surface, widespread hematogenous dissemination may occur [69]. Thus, invasive candidiasis is usually a consequence of a local or generalized defect in host defenses, together with an increased or abnormal colonization. Additionally, we found a decreasing trend in candidiasis rate during the study period. Again, as discussed above, this reduction could be related to a general improvement in intensive care that leads to fewer infections [24,70], more inclusive coding practices that increases the denominator and reduces the rate [47], and the impact of HCV treatments that stops liver disease in the vast majority of cured patients [19,48].

### Limitations and Strengths of the Study

This is a retrospective study that uses the Spanish MBDS (administrative database), and this entails a series of limitations of this type of study: (i) absence of significant information such as treatments, previous hospitalizations, prognostic scores (Model for End-stage Liver Disease (MELD), Child-Pugh Turcotte (CPT), Sequential Organ Failure Assessment (SOFA), or Acute Physiology and Chronic Health Evaluation (APACHE)), community-acquired or nosocomial nature of sepsis, therapeutic procedures, cause of hospital admission, etc. All this clinical data would give us information on the severity of the chronic hepatitis C and sepsis, information that could be used to stratify the study population and fit the regression models. In this way, we could know if sepsis only increased in patients with advanced liver disease, but not in non-cirrhotic patients, or only in those patients with an infection acquired in the hospital but not from the community. (ii) Some inaccuracies in coding may have occurred, which could cause a misclassification bias. In this regard, we have not evaluated the potential accuracy of the Spanish MBDS for sepsis diagnosis. Additionally, sepsis was defined according to the Angus algorithm [21], which is well-established in sepsis epidemiology [27,41]. The Angus algorithm offers a reasonable but also imperfect approach to classifying patients with sepsis [71]. Furthermore, we did not use the ICD-9 codes for sepsis (995.91 and 995.92) nor septic shock (785.52) because these codes were incorporated in Spain after 2003 and patients admitted with severe sepsis and septic shock are usually incompletely documented and under-coded [72]. This misclassification bias can make our estimates less accurate and under-estimated. (iii) A significant percentage of private hospitals did not have any data, and therefore, the rates may be underestimated. However, these centers are only a small percentage of the total hospitals in Spain. Additionally, we must highlight that the strength of our study is that data were analyzed nationwide, where the sample size was considerable. Our research performed on hospital admissions with chronic hepatitis C provides a general picture of the sepsis situation in the Spanish population, unlike studies in individual hospitals. (iv) MBDS data are anonymous and makes it difficult to identify patients who have been hospitalized several times in different hospitals, which can cause an overestimation of incidence and mortality rates. To this, we must add that we did not have the data of the reference population infected with HCV in Spain, which would have been the perfect population, but it was not possible because there was no record of HCV-infected patients between 2000–2015 in Spain. Furthermore, there was also not a sufficient number of published prevalence data to estimate the number of HCV-infected patients in Spain during the study period (2000–2015). (v) Our data were recorded before 2016 (more than five years ago), and our findings could not reflect the current characteristics and outcomes of sepsis. Since January 1, 2016, the ICD-10-CM is the reference classification for clinical coding and morbidity registration in the Spanish MBDS, replacing ICD-9-MC. We could extend the study until 2018 with data from ICD-10-CM, but we could introduce a bias that might invalidate our study [73]. (vi) DRGs were used to calculate costs, but it may not be a precise method due to different clinical conditions that may have widely varying costs. Despite this, the DRG system provides a uniform methodology to get hospital costs, and it may be applied to all hospitals of a National Health System. (vii) We were also unable to separate patients who presented with sepsis from those patients who developed sepsis during their hospital stay.

## 5. Conclusions

Sepsis, in chronic hepatitis C patients admitted to the hospital, has increased during the period of 2000–2015 and has been an increasing burden for the Spanish public health system. However, there has also been a significant reduction in lethality and LOHS during the study period. In addition, the most prevalent specific microorganisms have also changed in this period.

## Figures and Tables

**Table 1:** Epidemiological and clinical characteristics of hospital admissions of patients with chronic hepatitis C and sepsis in Spain (2000–2015).

	Entire Period	2000–2003	2004–2007	2008–2011	2012–2015	*p*-Value (*)
**No. of Hospital Admissions**	70,976	10,723	17,463	20,832	21,958	
**Gender (Male)**	44,825 (63.2%)	6781 (63.2%)	11,227 (64.3%)	13,102 (62.9%)	13,715 (62.5%)	0.006
**Age (Years)**	60 (16.7)	57.4 (17.9)	58.6 (17.2)	60.2 (16.5)	62.3 (15.5)	<0.001
≥50 Years	46,544 (65.6%)	6508 (60.7%)	10,501 (60.1%)	13,223 (63.5%)	16,312 (74.3%)	<0.001
**Substances of Abuse**						
Drugs	23,819 (33.6%)	3223 (30.1%)	5913 (33.8%)	7384 (35.4%)	7299 (33.2%)	<0.001
Alcohol	7881 (11.1%)	950 (8.8%)	1787 (10.2%)	2391 (11.5%)	2753 (12.5%)	<0.001
Tobacco	14,571 (20.5%)	1651 (15.4%)	3476 (19.9%)	4636 (22.2%)	4808 (21.9%)	<0.001
**Urgent Admission**	64,398 (90.7%)	9573 (89.3%)	15,760 (90.2%)	18,927 (90.8%)	20,138 (91.7%)	<0.001
**Surgical Condition**	7251 (10.2%)	1163 (10.8%)	1853 (10.6%)	2081 (9.9%)	2154 (9.8%)	0.001
**Charlson Index**	4.9 (2.9)	4.8 (2.7)	4.9 (2.8)	4.9 (2.9)	4.9 (2.9)	<0.001
**Liver Disease Severity**						
Non-Complicated Hepatitis C	39,077 (55.1%)	5764 (53.7%)	9690 (55.5%)	11,625 (55.8%)	11,998 (54.6%)	0.418
Compensated Cirrhosis	23,000 (32.4%)	3311 (30.9%)	5498 (31.5%)	6684 (32.1%)	7507 (34.2%)	<0.001
End-Stage Liver Disease	26,207 (36.9%)	4327 (40.3%)	6577 (37.6%)	7569 (36.3%)	7734 (35.2%)	<0.001
Hepatocellular Carcinoma	4222 (5.9%)	471 (4.4%)	901 (5.1%)	1295 (6.2%)	1555 (7.1%)	<0.001
Liver Transplant	1982 (2.8%)	217 (2.0%)	428 (2.4%)	637 (3.1%)	700 (3.2%)	<0.001
**Number of Acute Organ Dysfunction**						
Average	1.3 (0.7)	1.2 (0.6)	1.3 (0.6)	1.3 (0.7)	1.4 (0.7)	<0.001
1	54,622 (77%)	8852 (82.5%)	13,746 (78.7%)	15,970 (76.6%)	16,054 (73.1%)	<0.001
2	12,008 (16.9%)	1451 (13.5%)	2824 (16.2%)	3507 (16.8%)	4226 (19.2%)	<0.001
>2	4346 (6.1%)	420 (3.9%)	893 (5.1%)	1355 (6.5%)	1678 (7.6%)	<0.001
**Acute Organ Dysfunction**						
Cardiovascular	6721 (9.5%)	870 (8.1%)	1547 (8.8%)	2088 (10%)	2216 (10.1%)	<0.001
Hematologic	9487 (13.4%)	1251 (11.7%)	2204 (12.6%)	2649 (12.7%)	3383 (15.4%)	<0.001
Hepatic	14,542 (20.5%)	2931 (27.3%)	4167 (23.8%)	4220 (20.2%)	3224 (14.7%)	<0.001
Neurologic	3024 (4.3%)	494 (4.61%)	711 (4.1%)	835 (4%)	984 (4.5%)	0.898
Renal	21,264 (30%)	2752 (25.6%)	4559 (26.1%)	6064 (29.1%)	7889 (35.9%)	<0.001
Respiratory	35,023 (49.3%)	4579 (42.7%)	8568 (49.1%)	10,755 (51.6%)	11,121 (50.6%)	<0.001
Metabolic	3420 (4.8%)	294 (2.7%)	628 (3.6%)	1002 (4.8%)	1496 (6.8%)	<0.001
**Site of Infection**						
Respiratory	32,864 (46.3%)	4999 (46.6%)	8304 (47.5%)	9662 (46.4%)	9899 (45.1%)	<0.001
Digestive	23,528 (33.1%)	3984 (37.1%)	5964 (34.1%)	6795 (32.6%)	6785 (30.9%)	<0.001
Genitourinary	16,576 (23.4%)	2420 (22.6%)	3862 (22.1%)	4811 (23.1%)	5483 (24.9%)	<0.001

Values are expressed as absolute number (percentage) and mean (standard deviation). *p*-values: (*), the temporal trend was evaluated using the Extended Mantel Haenszel Chi-Square for linear trend for categorical variables and the Mann–Kendall Trend Test for continuous variables in Y values.

## Supplementary Materials

The following are available online at https://www.mdpi.com/2077-0383/9/6/1607/s1, Table S1: Summary of ICD-9-CM coding used for baseline comorbidities investigated in this study; Table S2: Temporal trend of the sepsis rate (regarding all hospital admissions with a diagnosis of chronic HCV infection, %) and the sepsis-related death (regarding chronic HCV-infected patients with hospital admission and sepsis, CFR, %) in Spain (2000–2015); Table S3: Temporal trend of the risk of sepsis (regarding all hospital admissions with a diagnosis of chronic HCV infection) and the risk of sepsis-related death (regarding chronic HCV-infected patients with hospital admission and sepsis) in Spain (2000–2015); Table S4: Temporal trend of the length of hospital stay and the cost in hospital admissions of patients with chronic hepatitis C and sepsis in Spain (2000–2015); Table S5: Temporal trend of microorganism specific rate (not including unknown) linked to sepsis and sepsis-related death in hospital admissions of patients with chronic hepatitis C and sepsis in Spain (2000–2015).

## Abbreviations

World Health Organization	(WHO)
Hepatitis C virus	(HCV)
Hepatocellular carcinoma	(HCC)
National Health Services	(NHS)
Intensive care unit	(ICU)
Cirrhosis-associated immune dysfunction	(CAID)
Case-fatality rate	(CFR)
Spanish Minimum Basic Data Set	(MBDS)
Ministry of Health, Consumption, and Social Welfare	(MHCSW)
International Classification of Diseases, 9th ed., Clinical Modification	(ICD-9-CM)
Length of hospital stay	(LOHS)
Supplementary Table	(ST)
Diagnosis-Related Groups	(DRG)
Odds ratio	(OR)
Adjusted odds ratio	(aOR)
Sustained virological response	(SVR)
Direct-acting antivirals	(DAAs)
Model for End-stage Liver Disease	(MELD)
Child-Pugh Turcotte	(CPT)
Sequential Organ Failure Assessment	(SOFA)
Acute Physiology and Chronic Health Evaluation	(APACHE)
