# Supplementary material for: Epidemiological Trend of Sepsis in Patients with Hospital Admissions Related to Hepatitis C in Spain (2000–2015): A Nationwide Study
Source: J Clin Med. 2020 May 26;9(6):1607. doi: 10.3390/jcm9061607 (PMC7355745; doi:10.3390/jcm9061607)
Supplement: Supplementary file 1 [file jcm-09-01607-s001.docx]

**Supplementary material:**

**Supplementary Table S1**. Summary of ICD-9-CM coding used for baseline comorbidities investigated in this study.

| **Description** | **Diagnosis codes (index or prior admissions)** |
| --- | --- |
| **HIV infection** | 042 or V08 |
| **Endocarditis** | 421.0, 421.1, 421.9 |
| **Abuse of alcohol and drugs** |  |
| Abuse of drugs | 292.x, 304.x, 305.x, and 965.0x |
| Abuse of alcohol | 305.0,303.0,303.9,291.0,291.1,291.2,291.3,291.4,291.5,291.8,291.9,571.0,571.1,571.2,571.3,425.5,535.3,357.5,265.2, V11.3,790.3,980.0 |
| Abuse of tobacco | 305.1, V15.82 |
| **Conditions influencing in health status** |  |
| Surgical conditions | V42, V45 |
| Trauma | E880* to E929*, E950 to E999* |
| **Charlson comorbidities [1]** |  |
| Myocardial infarction | 410.x, 412.x |
| Congestive heart failure | 428.x |
| Peripheral vascular disease | 443.9, 441.x, 785.4, V43.4, Procedure 38.48 |
| Cerebrovascular disease | 430.x–438.x |
| Dementia | 290.x |
| Chronic pulmonary disease | 490.x–505.x, 506.4 |
| Rheumatic disease | 710.0, 710.1, 710.4, 714.0–714.2, 714.81, 725.x |
| Peptic ulcer disease | 531.x–534.x |
| Mild liver disease | 571.2, 571.4–571.6 |
| Diabetes without chronic complication | 250.0–250.3, 250.7 |
| Diabetes with chronic complication | 250.4–250.6 |
| Hemiplegia or paraplegia | 344.1, 342.x |
| Renal disease | 582.x, 583–583.7, 585.x, 586.x, 588.x |
| Any malignancy, including lymphoma and leukemia, except malignant neoplasm of skin | 140.x–172.x, 174.x.–195.8, 200.x–208.x |
| Moderate or severe liver disease | 456.0–456.21, 572.2–572.8 |
| Metastatic solid tumor | 196.x–199.1 |
| AIDS/HIV | 042.x–044.x |
| **Organ failure** **[2-4]** |  |
| Cardiovascular | 427.5, 458.0, 458.8, 458.9, 785.5, 796.3 |
| Hematologic | 286.2, 286.6, 286.9, 287.3, 287.4, 287.5, 790.92 |
| Hepatic | 570, 572.2, 573.3, 573.4 |
| Neurologic | 293, 348.1, 348.3, 780.01, 780.09, 89.14 |
| Renal | 580, 580.0, 580.4, 580.8, 580.81, 580.89, 580.9, 584, 584.5, 584.6, 584.7, 584.8, 584.9, 586, 39.95 |
| Respiratory | 518.5, 518.8, 786.03, 799.1, 786.09, 96.7, 96.71, 96.72, 96.04, 93.90 |
| Metabolic | 276.2 |
| **Site of infection [5,6]** |  |
| Nervous | 013, 036, 091.81, 098.82, 320, 321, 321.1, 324, 325, 360, 376, 380.14, 383 |
| Circulatory | 093, 098.83 – 098.84, 036.4, 391.2, 420.99, 421 |
| Respiratory | 010.1, 011, 012, 018, 031.0, 032, 034, 098.6, 112.4, 114.0, 114.2, 115.15, 115.05, 115.95, 117.5, 117.3, 136.3, 461, 462, 463, 464, 465, 475, 480, 481, 482, 483, 485, 486, 487.0, 491.21, 494, 510, 513 |
| Digestive | 001, 002, 003, 004, 005, 008, 008.1, 008.2, 008.3, 008.4, 008.5, 009, 014, 129, 522.5, 522.7, 526.4, 527.3, 528.3, 540, 541, 542, 562.01, 562.03, 562.11, 562.13, 566, 567, 569.5, 569.61, 569.83, 572, 572.1, 575 |
| Genitourinary | 016, 098.17, 112.2, 590, 599, 601, 604, 614, 615, 616.3, 616.4 |
| Pregnancy | 634, 635, 636, 637, 638, 639, 646.6, 658.4, 670, 675.1 |
| Skin, soft tissue, or bone | 003.24, 015, 017, 031.1, 035, 036.82, 040.0, 095.5, 098.5, 681, 682, 683, 685, 686, 711, 728.86, 730 |
| Other | 790.7, 958.3, 996.6, 998.5, 999.3 |
| **Angus algorithm: bacterial and fungal codes [4]**. | 001, 002, 003, 004, 005, 008.0, 008.1, 008.2, 008.3, 008.4, 008.5, 009, 013, 018, 020, 021, 022, 023, 024, 025, 026, 027, 032, 033, 034, 035, 036, 037, 038, 039, 040, 041, 098, 100, 101, 112.0, 112.4, 112.5, 112.8, 114, 115, 116, 117, 118, 320, 321.0, 321.1, 324, 325, 360.0, 376.0, 380.14, 383.0, 420.99, 421, 461, 462, 463, 464, 465, 475, 481, 482, 485, 486, 491.21, 494, 510, 513, 522.5, 522.7, 526.4, 527.3, 528.3, 540, 541, 542, 562.01, 562.03, 562.11, 562.13, 566, 567, 569.5, 569.61, 569.83, 572.0, 572.1, 575.0, 590, 599.0, 601, 604, 614, 615, 616.3, 616.4, 634.0, 635.0, 636.0, 637.0, 638.0, 639.0, 646.6, 658.4, 670, 675.1, 681, 682, 683, 685.0, 686, 711.0, 728.86, 730, 790.7, 958.3, 996.6, 998.5, 999.3 |
| **Organism-specific that cause sepsis [7,8]** |  |
| Fungal infection |  |
| Candidiasis | 112.0, 112.4, 112.5, 112.8 |
| Aspergillosis | 117.3 |
| Zygomycosis | 117.7 |
| Gram-positive (+) | 038.1, 038.10, 038.11, 038.12, 038.19, 041.1, 041.11, 041.12, 041.10, 041.19, 482.41, and 482.42  038.0, 038.2, 041.0, 041.00, 041.01, 041.02, 041.03, 041.05, 041.09 and 041.2 |
| *Staphylococcus* | 038.1, 038.10, 038.11, 038.12, 038.19, 041.1, 041.11, 041.12, 041.10, 041.19, 482.41, and 482.42 |
| *Staphylococcus aureus* | 038.11, 038.12, 041.11, 041.12, 482.41, and 482.42 |
| Streptococcus | 038.0, 038.2, 041.0, 041.00, 041.01, 041.02, 041.03, 041.05, 041.09 and 041.2 |
| *Enterococcus* | 041.04 |
| Gram-negative (-) | 008.0, 008.00, 008.01, 008.02, 008.03, 008.04, 008.42, 008.09, 038.4, 038.40, 038.41, 038.42, 038.43, 038.44, 038.49, 041.3, 041.4, 041.5, 041.6, 041.7, 041.85, V01.83, 482.1, 482.0, 482.82, |
| *Gram*-negative NOS | 038.40 |
| *Escherichia coli* | 008.0, 008.00, 008.01, 008.02, 008.03, 008.04, 008.09, 038.42, 041.4, 482.82, V01.83 |
| *Pseudomonas* | 008.42, 038.43, 041.7, 482.1 |
| *Klebsiella* | 041.3, 482.0 |
| *Haemophilus influenzae* | 038.41, 041.5 |
| *Serratia* | 038.44 |

**References**

1. Gustot, T.; Felleiter, P.; Pickkers, P.; Sakr, Y.; Rello, J.; Velissaris, D.; Pierrakos, C.; Taccone, F.S.; Sevcik, P.; Moreno, C.*, et al.* Impact of infection on the prognosis of critically ill cirrhotic patients: Results from a large worldwide study. *Liver Int.* **2014**, *34*, 1496-1503.

2. Dombrovskiy, V.Y.; Martin, A.A.; Sunderram, J.; Paz, H.L. Rapid increase in hospitalization and mortality rates for severe sepsis in the united states: A trend analysis from 1993 to 2003. *Crit. Care Med.* **2007**, *35*, 1244-1250.

3. Shen, H.N.; Lu, C.L.; Yang, H.H. Epidemiologic trend of severe sepsis in taiwan from 1997 through 2006. *Chest* **2010**, *138*, 298-304.

4. Angus, D.C.; Linde-Zwirble, W.T.; Lidicker, J.; Clermont, G.; Carcillo, J.; Pinsky, M.R. Epidemiology of severe sepsis in the united states: Analysis of incidence, outcome, and associated costs of care. *Crit. Care Med.* **2001**, *29*, 1303-1310.

5. Esper, A.M.; Moss, M.; Lewis, C.A.; Nisbet, R.; Mannino, D.M.; Martin, G.S. The role of infection and comorbidity: Factors that influence disparities in sepsis. *Crit. Care Med.* **2006**, *34*, 2576-2582.

6. Wang, H.E.; Shapiro, N.I.; Angus, D.C.; Yealy, D.M. National estimates of severe sepsis in united states emergency departments. *Crit. Care Med.* **2007**, *35*, 1928-1936.

7. Ani, C.; Farshidpanah, S.; Bellinghausen Stewart, A.; Nguyen, H.B. Variations in organism-specific severe sepsis mortality in the united states: 1999-2008. *Crit. Care Med.* **2015**, *43*, 65-77.

8. Toyoda, N.; Chikwe, J.; Itagaki, S.; Gelijns, A.C.; Adams, D.H.; Egorova, N.N. Trends in infective endocarditis in california and new york state, 1998-2013. *JAMA* **2017**, *317*, 1652-1660.

**Table S2**. Temporal trend of the sepsis rate (regarding all hospital admissions with a diagnosis of chronic HCV infection, %) and the sepsis-related death (regarding chronic HCV-infected patients with hospital admission and sepsis, CFR, %) in Spain (2000–2015).

|  | **Sepsis** | | **Sepsis-related death** | |
| --- | --- | --- | --- | --- |
|  | **No.** | **Rate (95%CI)** |  |  |
| Whole follow-up | 70976 | 8.78% (8.71%; 8.84%) | 13915 | 19.61% (19.28%; 19.93%) |
| 2000-2003 | 10723 | 6.18% (6.06%; 6.3%) | 2358 | 21.99% (21.1%; 22.88%) |
| 2004-2007 | 17463 | 8.23% (8.11%; 8.35%) | 3530 | 20.21% (19.55%; 20.88%) |
| 2008-2011 | 20832 | 9.61% (9.48%; 9.74%) | 4040 | 19.39% (18.8%; 19.99%) |
| 2012-2015 | 21958 | 10.64% (10.5%; 10.78%) | 3987 | 18.16% (17.59%; 18.72%) |
| **P-values ^(^**^*)^ |  |  |  |  |
| 00-03 vs. 04-07 |  | **<0.001** |  | **0.010** |
| 00-03 vs. 08-11 |  | **<0.001** |  | **<0.001** |
| 00-03 vs. 12-15 |  | **<0.001** |  | **<0.001** |
| 04-07 vs. 08-11 |  | **<0.001** |  | 0.432 |
| 04-07 vs. 12-15 |  | **<0.001** |  | **<0.001** |
| 08-11 vs. 12-15 |  | **<0.001** |  | **0.019** |
| **P-values ^(^**^§)^ |  |  |  |  |
| Linear trend |  | **<0.001** |  | **<0.001** |

**Statistic**: Values were expressed as percentages. (*), differences between groups by Chi-Square test. (§) linear trend from 2000-2003 to 2012-2015 by the Extended Mantel Haenszel Chi-Square. Statistically significant differences are shown in bold.

**Abbreviations**: HCV, hepatitis C virus, CFR, case fatality rate.

**Table S3**. Temporal trend of the risk of sepsis (regarding all hospital admissions with a diagnosis of chronic HCV infection) and the risk of sepsis-related death (regarding chronic HCV-infected patients with hospital admission and sepsis) in Spain (2000–2015).

|  | **Univariate** | | **Multivariate** | |
| --- | --- | --- | --- | --- |
| **A) Sepsis** | **OR (95%CI)** | **p-value** | **aOR (95%CI)** | **p-value** |
| **Calendar period** |  |  |  |  |
| 2000-2003 | Ref. |  | Ref. |  |
| 2004-2007 | 0.91 (0.89; 0.93) | **<0.001** | 1.31 (1.28; 1.35) | **<0.001** |
| 2008-2011 | 1.15 (1.13; 1.17) | **<0.001** | 1.49 (1.46; 1.53) | **<0.001** |
| 2012-2015 | 1.34 (1.32; 1.36) | **<0.001** | 1.58 (1.54; 1.62) | **<0.001** |
| **Gender (male)** | 1.01 (0.99; 1.02) | 0.417 | 1.06 (1.05; 1.08) | **<0.001** |
| **Age ≥50 years** | 1.47 (1.45; 1.5) | **<0.001** | 1.55 (1.53; 1.58) | **<0.001** |
| **Urgent admission** | 5.17 (5.04; 5.31) | **<0.001** | 4.51 (4.4; 4.63) | **<0.001** |
| **Surgical condition** | 0.91 (0.88; 0.93) | **<0.001** | 0.82 (0.8; 0.84) | **<0.001** |
| **Charlson index** | 1.11 (1.1; 1.11) | **<0.001** | 1.09 (1.09; 1.1) | **<0.001** |
| **Liver disease severity** |  |  |  |  |
| Non-complicated hepatitis C | Ref. |  | Ref. |  |
| Compensated cirrhosis | 1.42 (1.40; 1.45) | **<0.001** | 0.99 (0.98; 1.02) | 0.967 |
| End-stage liver disease | 1.83 (1.8; 1.86) | **<0.001** | 1.33 (1.3; 1.36) | **<0.001** |
|  |  |  |  |  |
| **B) Sepsis-related death** | **OR (95%CI)** | **p-value** | **aOR (95%CI)** | **p-value** |
| **Calendar period** |  |  |  |  |
| 2000-2003 | Ref. | - | Ref. | - |
| 2004-2007 | 1.05 (1.01; 1.1) | **0.020** | 0.81 (0.76; 0.86) | **<0.001** |
| 2008-2011 | 0.98 (0.94; 1.02) | 0.359 | 0.71 (0.66; 0.75) | **<0.001** |
| 2012-2015 | 0.87 (0.84; 0.91) | **<0.001** | 0.56 (0.53; 0.60) | **<0.001** |
| **Gender (male)** | 0.96 (0.93; 1) | 0.063 | 0.91 (0.87; 0.95) | **<0.001** |
| **Age** ≥50 years | 1.36 (1.3; 1.41) | **<0.001** | 1.61 (1.53; 1.69) | **<0.001** |
| **Urgent admission** | 0.97 (0.91; 1.04) | 0.408 | 1.03 (0.96; 1.10) | 0.460 |
| **Surgical condition** | 0.78 (0.73; 0.83) | **<0.001** | 0.93 (0.87; 0.99) | **0.039** |
| **Charlson index** | 1.05 (1.04; 1.06) | **<0.001** | 1.06 (1.05; 1.06) | **<0.001** |
| **Length of stay (>15 days)** | 0.77 (0.74; 0.8) | **<0.001** | 0.95 (0.91; 0.99) | **0.02** |
| **Liver disease severity** |  |  |  |  |
| Non-complicated CHC | Ref. | - | Ref. | - |
| Compensated cirrhosis | 1.91 (1.84; 1.98) | **<0.001** | 1.40 (1.33; 1.48) | **<0.001** |
| End-stage liver disease | 1.94 (1.87; 2.01) | **<0.001** | 1.28 (1.2; 1.37) | **<0.001** |
| **No. of acute organ dysfunction** |  |  |  |  |
| 1 | Ref. | - | Ref. | - |
| 2 | 2.43 (2.32; 2.53) | **<0.001** | 3.24 (3.09; 3.4) | **<0.001** |
| >2 | 9.16 (8.58; 9.78) | **<0.001** | 13.37 (12.5; 14.3) | **< 0.001** |
| **Organism-Specific Sepsis** |  |  |  |  |
| *Staphylococcus aureus* | 1.44 (1.33; 1.57) | **<0.001** | 1.19 (1.08; 1.31) | **<0.001** |
| *Enterococcus* | 0.96 (0.85; 1.08) | 0.490 | 0.93 (0.82; 1.05) | 0.247 |
| *Escherichia coli* | 0.99 (0.92; 1.05) | 0.669 | 0.93 (0.86; 1.01) | 0.088 |
| *Pseudomonas* | 0.99 (0.90; 1.09) | 0.955 | 1.06 (0.95; 1.18) | 0.295 |
| *Klebsiella* | 0.87 (0.76; 0.99) | **0.041** | 0.89 (0.77; 1.03) | 0.113 |
| **Site of infection** |  |  |  |  |
| Digestive | 1.81 (1.75; 1.88) | **<0.001** | 0.95 (0.89; 1.02) | 0.133 |
| Genitourinary | 0.69 (0.65; 0.72) | **<0.001** | 0.53 (0.50; 0.56) | **<0.001** |
| Respiratory | 0.77 (0.75; 0.80) | **<0.001** | 0.81 (0.77; 0.85) | **<0.001** |

**Statistic**: Values were expressed as odds ratios (OR) and 95% of confidence intervals (95%CI). P-values were calculated by logistic regression analysis. Statistically significant differences are shown in bold.

**Abbreviations**: HCV, hepatitis C virus; aOR, adjusted odds ratio; 95%CI, 95% of confidence interval.

**Table S4**. Temporal trend of the length of hospital stay and the cost in hospital admissions of patients with chronic hepatitis C and sepsis in Spain (2000–2015).

|  | **Hospital stay**  **(days)** | **Cost/patient**  **(x10^3^ euros)** | **National expenditure**  **(x10^6^ euros)** |
| --- | --- | --- | --- |
|  | **Mean (95%CI)** | **Mean (95%CI)** | **Mean** |
| Whole follow-up | 15.3 (15.2; 15.5) | 9,089 (8961; 9217) | 645.1 |
| 2000-2003 | 16.9 (16.5; 17.2) | 7,196 (6942; 7450) | 77.1 |
| 2004-2007 | 16.3 (16.0; 16.6) | 8,568 (8348; 8793) | 149.6 |
| 2008-2011 | 15.3 (15.1; 15.6) | 10,070 (9793; 10347) | 209.7 |
| 2012-2015 | 13.9 (13.7; 14.2) | 9,497 (9261; 9733) | 208.5 |
| **P-values** ^(*)^ |  |  |  |
| 00-03 vs. 04-07 | 0.059 | **<0.001** |  |
| 00-03 vs. 08-11 | **<0.001** | **<0.001** |  |
| 00-03 vs. 12-15 | **<0.001** | **<0.001** |  |
| 04-07 vs. 08-11 | **<0.001** | **<0.001** |  |
| 04-07 vs. 12-15 | **<0.001** | **<0.001** |  |
| 08-11 vs. 12-15 | **<0.001** | **0.004** |  |
| **P-values** ^(§)^ |  |  |  |
| Linear trend | **<0.001** | **<0.001** | **<0.001** |

**Statistic**: Values expressed as mean (95%CI). P-values: (*), differences between groups by ANOVA test. (§) linear trend from 2000-2003 to 2012-2015 by the Mann-Kendall Trend Test. Statistically significant differences are shown in bold.

**Abbreviations**: HCV, hepatitis C virus.

**Table S5**. Temporal trend of microorganism specific rate (not including unknown) linked to sepsis and sepsis-related death in hospital admissions of patients with chronic hepatitis C and sepsis in Spain (2000–2015).

|  | **Entire period** | **2000-2003** | **2004-2007** | **2008-2011** | **2012-2015** | **p-value (*)** |
| --- | --- | --- | --- | --- | --- | --- |
| **Sepsis-related admission** |  |  |  |  |  |  |
| Gram-positive (+) | 6917 (9.7%) | 1066 (9.94%) | 1728 (9.9%) | 2098 (10.07%) | 2025 (9.22%) | **0.027** |
| Staphylococci | 4584 (6.5%) | 776 (7.24%) | 1178 (6.75%) | 1319 (6.33%) | 1311 (5.97%) | **<0.001** |
| Coagulase-negative staphylococci | 1553 (2.2%) | 276 (2.57%) | 411 (2.35%) | 432 (2.07%) | 434 (1.97%) | **<0.001** |
| *Staphylococcus aureus* | 3031 (4.3%) | 500 (4.66%) | 767 (4.39%) | 887 (4.26%) | 877 (3.99%) | **0.003** |
| Streptococci | 2456 (3.5%) | 309 (2.88%) | 585 (3.35%) | 807 (3.87%) | 755 (3.44%) | **0.007** |
| Enterococci | 1886 (2.7%) | 217 (2.02%) | 409 (2.34%) | 553 (2.65%) | 707 (3.22%) | **<0.001** |
| Gram-negative (-) | 12045 (17%) | 1541 (14.37%) | 2862 (16.39%) | 3866 (18.56%) | 3776 (17.2%) | **<0.001** |
| *Escherichia coli* | 5740 (8.1%) | 858 (8%) | 1536 (8.8%) | 1952 (9.37%) | 1394 (6.35%) | **<0.001** |
| *Pseudomonas* | 2653 (3.7%) | 305 (2.84%) | 615 (3.52%) | 830 (3.98%) | 903 (4.11%) | **<0.001** |
| *Klebsiella* | 1559 (2.2%) | 107 (1%) | 287 (1.64%) | 512 (2.46%) | 653 (2.97%) | **<0.001** |
| Candidiasis | 5961 (8.4%) | 1072 (10%) | 1623 (9.29%) | 1685 (8.1%) | 1581 (7.2%) | **<0.001** |
| **Sepsis-related death** |  |  |  |  |  |  |
| Gram-positive (+) | 1630 (11.7%) | 320 (13.57%) | 419 (11.87%) | 459 (11.36%) | 432 (10.84%) | **0.001** |
| Staphylococci | 1138 (8.2%) | 243 (10.31%) | 314 (8.9%) | 300 (7.43%) | 281 (7.05%) | **<0.001** |
| Coagulase-negative staphylococci | 359 (2.6%) | 81 (3.43%) | 97 (2.74%) | 92 (2.27%) | 89 (2.23%) | **0.002** |
| *Staphylococcus aureus* | 779 (5.6%) | 162 (6.87%) | 217 (6.15%) | 208 (5.15%) | 192 (4.82%) | **<0.001** |
| Streptococci | 522 (3.8%) | 81 (3.44%) | 116 (3.29%) | 166 (4.11%) | 159 (3.99%) | 0.086 |
| Enterococci | 358 (2.6%) | 40 (1.7%) | 73 (2.07%) | 107 (2.65%) | 138 (3.46%) | **<0.001** |
| Gram-negative (-) | 2381 (17.1%) | 383 (16.24%) | 571 (16.18%) | 738 (18.27%) | 689 (17.28%) | 0.086 |
| *Escherichia coli* | 1113 (8%) | 193 (8.18%) | 286 (8.1%) | 353 (8.74%) | 281 (7.05%) | 0.138 |
| *Pseudomonas* | 519 (3.7%) | 93 (3.94%) | 127 (3.6%) | 163 (4.03%) | 136 (3.41%) | 0.444 |
| *Klebsiella* | 274 (2%) | 20 (0.85%) | 51 (1.44%) | 88 (2.18%) | 115 (2.88%) | **<0.001** |
| Candidiasis | 988 (7.1%) | 196 (8.31%) | 281 (7.96%) | 275 (6.81%) | 236 (5.92%) | **<0.001** |

**Statistic:** Values are expressed as absolute number (percentage). (*), linear trend from 2000-2003 to 2012-2015 by the Extended Mantel Haenszel Chi-Square. Statistically significant differences are shown in bold. **Abbreviations**: HCV, hepatitis C virus.
